# Utilization of Intravenous Ribavirin Among Reproductive Age Adults in 2010–2017: A Population-Based Study in the Yinzhou District, Ningbo City of China

**DOI:** 10.3389/fpubh.2021.678785

**Published:** 2021-09-17

**Authors:** Hailong Li, Houyu Zhao, Hongbo Lin, Peng Shen, Cuili Liu, Siyan Zhan

**Affiliations:** ^1^Department of Epidemiology and Biostatistics, School of Public Health, Peking University, Beijing, China; ^2^Yinzhou District Center for Disease Control and Prevention, Ningbo, China; ^3^Center for Drug Revaluation, National Medical Products Administration (NMPA), Beijing, China

**Keywords:** intravenous ribavirin, reproductive age, drug utilization, rational drug use, population-based study

## Abstract

**Background:** Intravenous (IV) ribavirin is not approved in US and European Union, but it is authorized in China. Significant teratogenic and embryocidal effects of ribavirin have been found in almost all animal studies, it is critical to investigate the prevalence and trends of the utilization of IV ribavirin among reproductive age population.

**Objective:** To evaluate the prevalence and trends of IV ribavirin use among reproductive-age population in 2010–2017.

**Methods:** The study design of our study is retrospective cross-sectional study based on healthcare database. We identified and extracted the data of residents aged 18–44 years by using Yinzhou healthcare information database at 21 January, 2018. A cohort of IV ribavirin users were identified through outpatient prescription records in 3 general hospitals and 24 community health centers from 2010 to 2017. We reported the number, proportion, and prevalence of the exposure to IV ribavirin stratified by sex, age, marital status, education level, occupation, hospital level, calendar year, diagnosis, and dosage. The overall trends of IV ribavirin use, and the trends in different levels of hospital and common diagnoses were further analyzed and described.

**Result:** During the study period, the prevalence of IV ribavirin use among reproductive-age adults was 6.02% (48,287/801,667). Relatively higher prevalence were found in adults aged 40–44 (8.04%, 95% CI: 7.90–8.17), unmarried patients (8.91%, 95% CI: 8.74–9.08), and who had more than 9 years of education (6.82%, 95% CI: 6.74–6.90). Compared to secondary and tertiary hospitals, IV ribavirin was more likely to be dispensed in primary hospitals (19.44%, 95% CI: 19.28–19.61). The most common diagnoses were acute upper respiratory infections (AURIs), accounting for 80% of the patients exposed to IV ribavirin. For patients with AURIs, the prevalence of IV ribavirin was nearly 30%. Overall, the prevalence of IV ribavirin use decreased from 1.72% in 2010 to 0.24% in 2017.

**Conclusion:** We found IV ribavirin was mainly used for AURIs which suggested that a large amount of IV ribavirin use was probably inappropriate. The prevalence was decreasing by 87% over the past 8 years, and we encourage clinicians and pharmacists to continually avoid inappropriate use of IV ribavirin.

## Introduction

Ribavirin is a kind of antiviral drug which has direct antiviral activity in tissue culture against many RNA viruses. Ribavirin increases the mutation frequency in the genomes of several RNA viruses and Ribavirin triphosphate inhibits HCV polymerase in a biochemical reaction (1). Oral ribavirin used in combination with interferon alfa-2b (non-pegylated) for treatment of chronic hepatitis C (CHC) and ribavirin aerosol used for the treatment of severe lower respiratory tract infections due to respiratory syncytial virus (RSV) in children have been approved by the United States Food and Drug Administration (FDA) (1–7). Because lack of evidence proves the efficacy and safety of intravenous (IV) ribavirin, it is not approved in most developed countries and regions (e.g., North America, European Union, and Australia) (2). However, in China, it is authorized for the treatment of patients with pneumonia and bronchitis due to RSV. Human RSV is the major cause of many respiratory tract diseases among infants and young children (8). For adults, a study in China shows that among 9,871 patients who went to the hospital because of acute respiratory infections, only 95 (1%) patients were detected positive for RSV by RT-PCR analysis (9). Moreover, RSV infections in adults are typically mild, transient and do not require hospitalization or antiviral treatment (10, 11). Thus, it is probably inappropriate to prescribe IV ribavirin to adults with acute respiratory infections.

Ribavirin has been assigned to pregnancy category X by the FDA which means that its use is contraindicated in pregnant women. Nearly all animal studies have demonstrated its significant teratogenic and embryocidal effects. In addition, ribavirin has a multiple-dose half-life of 12 days, and so it may persist in non-plasma compartments for as long as 6 months (12, 13). Therefore, female patients receiving ribavirin and the partners of male patients taking ribavirin therapy must avoid pregnancy, using at least two reliable types of contraception, during therapy and at least 6 months after completion of treatment (14, 15). In China, the IV ribavirin instructions clearly state that ribavirin is contraindicated in pregnant women, lactating women, and women who may be pregnant. Therefore, it is at a high risk of using IV ribavirin for men and women in reproductive age.

Despite that IV ribavirin had been approved in China for many years, little is known about the prevalence and trends of its use among adults. Quantifying the use of IV ribavirin among adults in reproductive age is a crucial step toward evaluating its risk-benefit. Thus, we aimed to investigate the utilization of IV ribavirin in adults aged 18–44 years old using Yinzhou healthcare information database from 2010 to 2017 in this observational study.

## Materials and Methods

### Data Resources

Yinzhou is a district in Ningbo, an eastern coastal city of China, with nearly 1.5 million residents in 2015. In 2005, the local health authority rolled out a Health Information System, which has collected and administrated the healthcare information of the residents since then. By 2010, this system covered all the hospitals in Yinzhou. Moreover, it integrated electronic medical records (EMRs) of hospitals, public health surveillance and disease management among communities. The administrative healthcare database stored all the data, including outpatient and inpatient records, prescribing information, and general demographic characteristics. The outpatient and inpatient records comprised hospital code, patient code, date of diagnosis, diagnosis name and diagnosis code (10th revision of the International Classification of Diseases and Related Health Problems, ICD-10). Prescribing information consisted of prescription code, prescription date, and product name, brand, generic name, and dosage form and dosage frequency of drugs. All the data from patients was anonymized and no personal details were shared with health researchers for privacy protection. This database has been used and described in previous studies (16–20). This study was approved by the ethical review board of Peking University Health Science Center (IRB00001052-18013). Informed consent was not required owing to the use of anonymized routine data. We extracted the data was at January 21, 2018.

### Study Population

**Figure 1:**
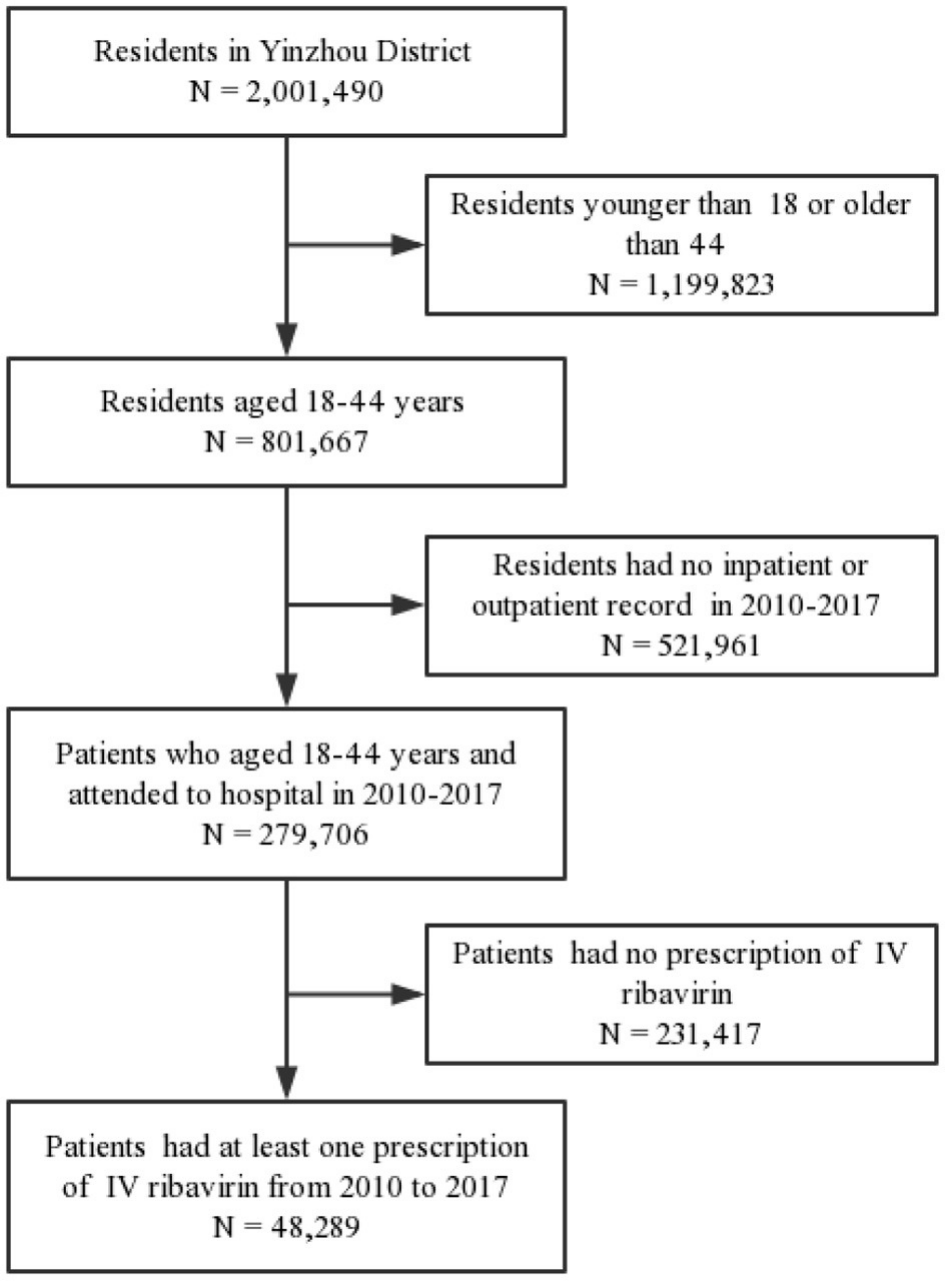
Flowchart of identifying patients who ever used IV ribavirin.

The study design of our study is retrospective cross-sectional study based on healthcare database. We first identified the eligible residents by using the health archives in the Yinzhou healthcare information database. The inclusion criteria is the residents aged 18–44 years old who have a high probability to be pregnant or be the partners of pregnant women. We define the residents who met this inclusion criteria as general population. As shown in Figure 1, the residents aged 18–44 years old when they went to the hospital from 2010 to 2017 were included *via* electronic medical records of 28 hospitals, including 3 general hospitals (2 tertiary hospitals, 1 secondary hospital) and 24 primary healthcare facilities. Furthermore, we excluded the patients who had no prescription records of IV ribavirin during the study period. By using the database, we extracted the information of patients ID, sex, birthday, marital status, education level, occupation, diagnosis code (ICD-10), diagnosis name, hospital level, prescription date, and generic name and dose of drugs.

### Statistical Analysis

The number of IV ribavirin prescriptions and the number of patients who had ever exposed to ribavirin were reported among the reproductive age patients. We calculated the overall prevalence and its Wald 95% confidence intervals of IV ribavirin exposure where the denominator was the general population in reproductive age.

Additionally, prevalence estimates for IV ribavirin exposure were calculated, stratified by characteristics including sex, age, marital status, educational level, and occupation. We also investigated the prevalence of IV ribavirin among patients in different levels of hospitals with the numerator reflecting the total number of patients exposed to IV ribavirin in a given level of hospital and the denominator consisting of the total number of patients in the same level of the hospital.

The most common corresponding diagnoses (ICD-10) of IV ribavirin users were reported with a specific prevalence. In this case, the numerator was the number of IV ribavirin users with a given diagnosis and the denominator was the total number of patients with the same diagnosis.

The proportions of IV ribavirin overdose were evaluated for both single dose and daily dose. According to the package inserts of the IV ribavirin (500 mg each time, 2 times per day), we stratified the single dose by 500 mg and the daily dose by 1,000 mg.

Finally, we plotted the trends in the use of IV ribavirin from 2010 to 2017. The year-specific prevalence for IV ribavirin exposure in different hospitals and diagnoses were described. In these estimates, the numerator was the number of IV ribavirin users in a given hospital/with a given diagnosis for each year and the denominator was the total number of patients in the same hospital/with the same diagnosis for a specific year. Furthermore, we conducted a time series analysis to test the change of IV ribavirin use.

## Results

As the general population in our study, there were 801,667 residents of reproductive age in Yinzhou from 2010 to 2017. During the study period, our study cohort comprised 48,289 patients aged 18–44 years with at least one prescription record of IV ribavirin. Overall, the prevalence of IV ribavirin was 6.02% (48,287/801,667).

Table 1 provides the baseline characteristic of the general population and patients exposed to IV ribavirin in this study. The numbers of male and female patients who had ever received IV ribavirin were nearly equal, but the prevalence of IV ribavirin was higher among males than that among females (6.33%, 95% CI: 6.25–6.41 vs. 5.75%, 95% CI: 5.68–5.82, *P* < 0.001). The prevalence of IV ribavirin (8.04%, 95% CI: 7.90–8.17) was highest in patients aged 40–44 years old (*P* < 0.001). Approximately 70% of patients of the study cohort were married, followed by 20% unmarried, 10% others. The unmarried patients were the most likely to be treated with IV ribavirin (8.91%, 95% CI: 8.74–9.08, *P* < 0.001). Patients with <9 years of education were more likely to be exposed to IV ribavirin compared to patients with more than 9 years of education (6.82%, 95% CI: 6.74–6.90 vs. 5.32%, 95% CI: 5.25–5.39, *P* < 0.001). The prevalence of using IV ribavirin were highest (16.93%) among students, followed by 10.15% among farmer, and 9.87% among the unemployed (*P* < 0.001).

**Table 1:** Baseline characteristics of general population and patients exposed to the IV ribavirin.

**Characteristics**	**General population**	**Ribavirin user**	**Proportion (%)**	**PR (95% CI)**	***P***
**Sex**					<0.001
Male	378,782	23,969	49.64	6.33 (6.25–6.41)	
Female	422,885	24,318	50.36	5.75 (5.68–5.82)	
**Age**					<0.001
18–24	195,723	9,372	19.41	4.79 (4.69–4.88)	
25–29	152,251	9,387	19.44	6.17 (6.04–6.29)	
30–34	143,073	10,800	22.37	7.55 (7.41–7.69)	
35–39	153,259	10,353	21.44	6.76 (6.63–6.88)	
40–44	157,361	12,644	26.19	8.04 (7.90–8.17)	
**Marital status**					<0.001
Unmarried	110,067	9,808	20.31	8.91 (8.74–9.08)	
Married	610,331	33,765	69.93	5.53 (5.47–5.59)	
Divorced	5,388	360	0.75	6.68 (6.01–7.35)	
Unknown	75,881	4,354	9.02	5.74 (5.57–5.90)	
**Education level**					<0.001
<9 years	376,322	25,666	53.15	6.82 (6.74–6.90)	
≥9 years	425,345	22,621	46.85	5.32 (5.25–5.39)	
**Occupation**					<0.001
Workers	240,898	14,499	30.03	6.02 (5.92–6.11)	
Students	22,250	3,766	7.80	16.93 (16.43–17.42)	
Service staff	110,916	4,892	10.13	4.41 (4.29–4.53)	
Civil servant	73,367	4,522	9.36	6.16 (5.99–6.34)	
Unemployed	29,523	2,915	6.04	9.87 (9.53–10.21)	
Farmer	66,220	6,724	13.93	10.15 (9.92–10.38)	
Others	189,099	7,243	15.00	3.83 (3.74–3.92)	
Unknown	69,394	3,726	7.72	5.3 7 (5.20-5.54)	

*Proportion: the denominator was the total number of IV ribavirin users (N = 48,289)*.

*PR, prevalence rate, for which the denominator was the total number of general population*.

As shown in Table 2, primary hospitals were more likely to dispense IV ribavirin (19.44%, 95% CI: 19.28–19.61, *P* < 0.001) than secondary hospitals (14.87%, 95% CI: 14.36–15.40) and tertiary hospitals (10.73%, 95% CI: 10.41–11.05).

**Table 2:** Prevalence of ribavirin users in different level of hospital.

**Hospital level**	**Total patients**	**Ribavirin users**	**Proportion (%)**	**PR (95% CI)**
Primary	218,926	42,567	88.15	19.44 (19.28–19.61)
Secondary	18,155	2,700	5.59	14.87 (14.36–15.40)
Tertiary	35,872	3,848	7.97	10.73 (10.41–11.05)

*Proportion: the denominator was the total number of IV ribavirin users (N = 48,289)*.

*PR, prevalence rate, where the denominator was the total number of patients in specific level of hospital*.

The most common diagnosis was acute upper respiratory infections (AURIs), presenting in 80% of diagnosis records of the patients exposed to IV ribavirin (Table 3). Among outpatients whose first diagnosis was AURIs, the prevalence of IV ribavirin was nearly 30%. There were almost 7.23% of IV ribavirin users had other infectious diseases (other and unspecified infectious diseases) as the first diagnosis. The prevalence of IV ribavirin were 25, 24.14, 21.08 14.80, and 14.52% among patients who had a diagnosis of viral hemorrhagic fevers, other respiratory disorders, other infectious diseases, viral infections characterized by skin and mucous membrane lesions, and fever of other and unknown origin, respectively.

**Table 3:** Prevalence of IV ribavirin users among patients with different diagnoses.

**Diagnosis**	**All patients**	**Ribavirin users (%)**	**PR (%)**	**95% CI**
Acute upper respiratory infections^a^	130,079	38,424 (79.57)	29.54	29.29–29.79
Other infectious disease^b^	16,558	3,491 (7.23)	21.08	20.46–21.71
Other respiratory disorders^c^	8,992	2,171 (4.50)	24.14	23.26–25.04
Other diseases of upper respiratory tract^d^	37,750	1,977 (4.09)	5.24	5.01–5.47
Disorders of conjunctiva	22,859	1,154 (2.39)	5.05	4.77–5.34
Other acute lower respiratory infections^e^	17,316	1,065 (2.21)	6.15	5.80–6.52
Viral infections characterized by skin and mucous membrane lesions^f^	6,884	1,019 (2.11)	14.80	13.97–15.66
Fever of other and unknown origin	5,146	747 (1.55)	14.52	13.56–15.51
Chronic lower respiratory diseases	17,907	558 (1.16)	3.12	2.87–3.38
Keratitis	8,178	264 (0.55)	3.23	2.86–3.63
Other and unspecified non-infective gastroenteritis and colitis	24,909	140 (0.29)	0.56	0.47–0.66
Stomatitis and related lesions	7,753	78 (0.16)	1.01	0.80–1.25
Viral hemorrhagic fevers	4	1 (0.00)	25.00	0.63–80.59
Other diagnoses^g^	253,901	3,951 (8.18)	1.56	1.51–1.61

*PR, prevalence rate, where the denominator was the total number of patients with specific diagnosis*.

a *ICD10: J00~J06, including acute nasopharyngitis (common cold), acute sinusitis, acute pharyngitis, acute tonsillitis, acute laryngitis and tracheitis, acute obstructive laryngitis (croup) and epiglottitis and acute upper respiratory infections of multiple and unspecified sites*.

b *ICD10: B99, including other infectious disease, Unspecified infectious disease*.

c *ICD10: J98, including diseases of bronchus, not elsewhere classified, pulmonary collapse, interstitial emphysema, compensatory emphysema, other disorders of lung, diseases of mediastinum, etc*.

d *ICD10: J30~J39, including vasomotor and allergic rhinitis, chronic rhinitis, nasopharyngitis and pharyngitis, chronic sinusitis, peritonsillar abscess etc*.

e *ICD10: J20~J22, other acute lower respiratory infections, acute bronchitis, acute bronchiolitis, unspecified acute lower respiratory infection, etc*.

f *ICD10: B00~B09, viral infections characterized by skin and mucous membrane lesion, etc*.

g *Including gastritis and duodenitis, dyspepsia, rashes and other non-specific skin eruptions, cough, etc*.

Table 4 demonstrates the numbers and proportions of users and prescriptions in different doses. There were 89,225 prescriptions of IV ribavirin with a single dose ≤ 500 mg, accounting for 86% of the total number of IV ribavirin exposure. Further, the proportion of prescriptions with daily diose ≤ 1,000 mg of IV ribavirin was 99.28% (*n* = 103,274).

**Table 4:** Proportions of IV ribavirin prescriptions in different doses.

**Dosage (mg)**	***N***	**Proportion (%)**	**95% CI**
**Single dose**
≤500	89,225	85.78	85.56–85.99
>500	14,796	14.22	14.01–14.44
Total	104,021	–	–
**Daily dose**
≤1,000	103,274	99.28	99.23–99.33
>1,000	747	0.72	0.67–0.77
Total	104,021	–	–

**Figure 2:**
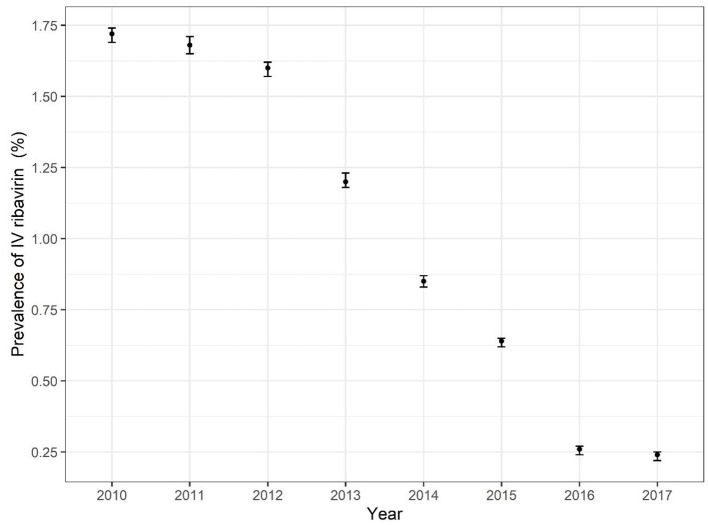
Prevalence of Use of IV ribavirin by Calendar Year among general population in reproductive age.

Figure 2 and Appendix Table 1 reveal the trends of IV ribavirin use among the general population aged 18–44 over the 8 years. The prevalence of IV ribavirin users decreased from 1.72% (95% CI: 1.69–1.74) in 2010 to 0.24% (95% CI: 0.22–0.25) in 2017 (*P* < 0.001). For the prescription of IV ribavirin, the prevalence decreased from 6.4% in 2010 to 0.56% in 2017 (*P* < 0.001). Changes of IV ribavirin prevalence among patients and prescriptions are illustrated in Figure 2 and Appendix Table 2.

**Figure 3:**
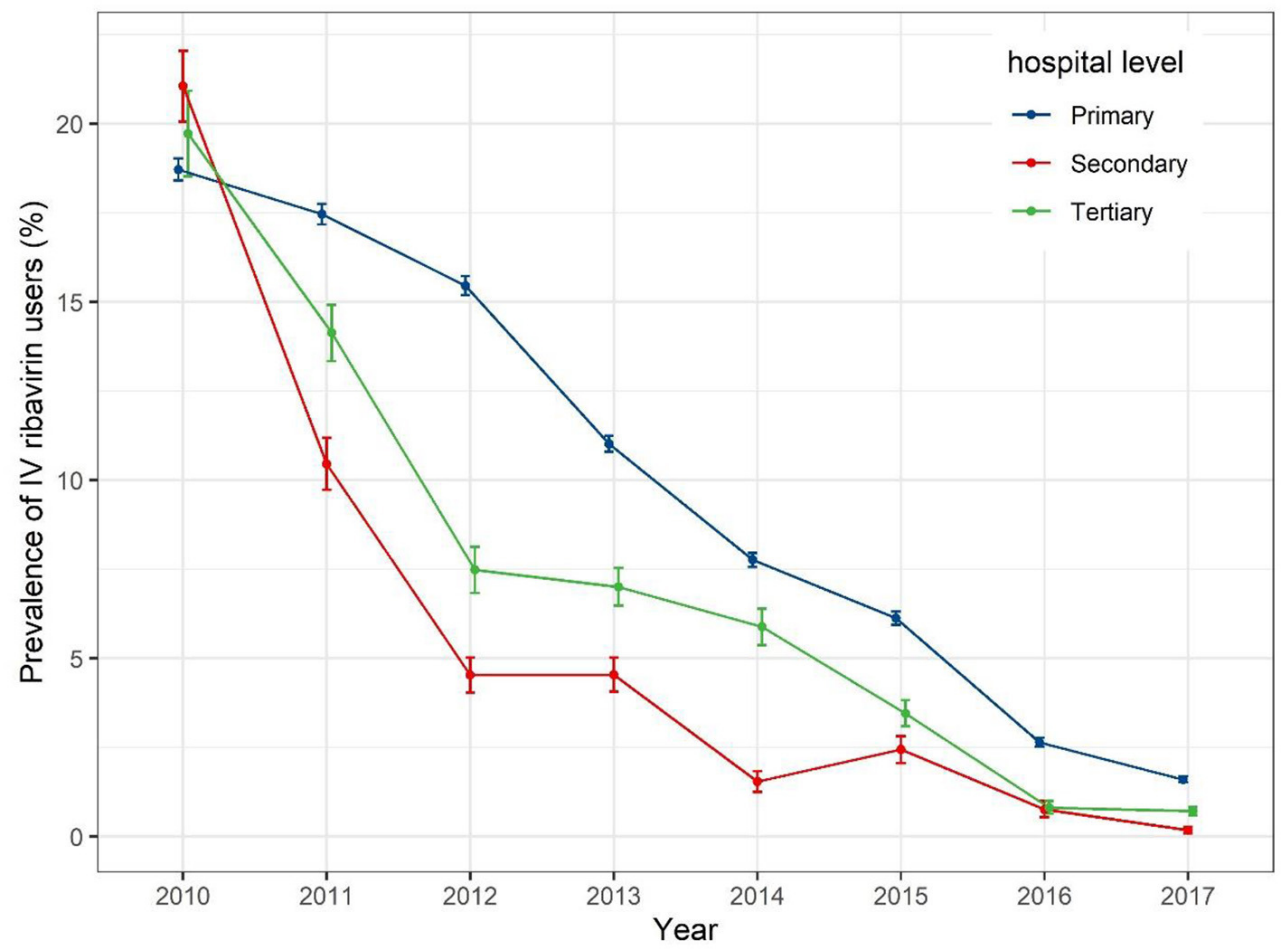
Prevalence of use of IV ribavirin from 2010 to 2017 stratified by hospital level.

When IV ribavirin prescription prevalence is stratified by hospital level (Figure 3), a gradual decrease was seen for all levels of hospitals, from around 20% in 2010 to around 2% in 2017. The prevalence of dispensed IV ribavirin decreased sharply from 2010 to 2012 for secondary and tertiary hospitals.

**Figure 4:**
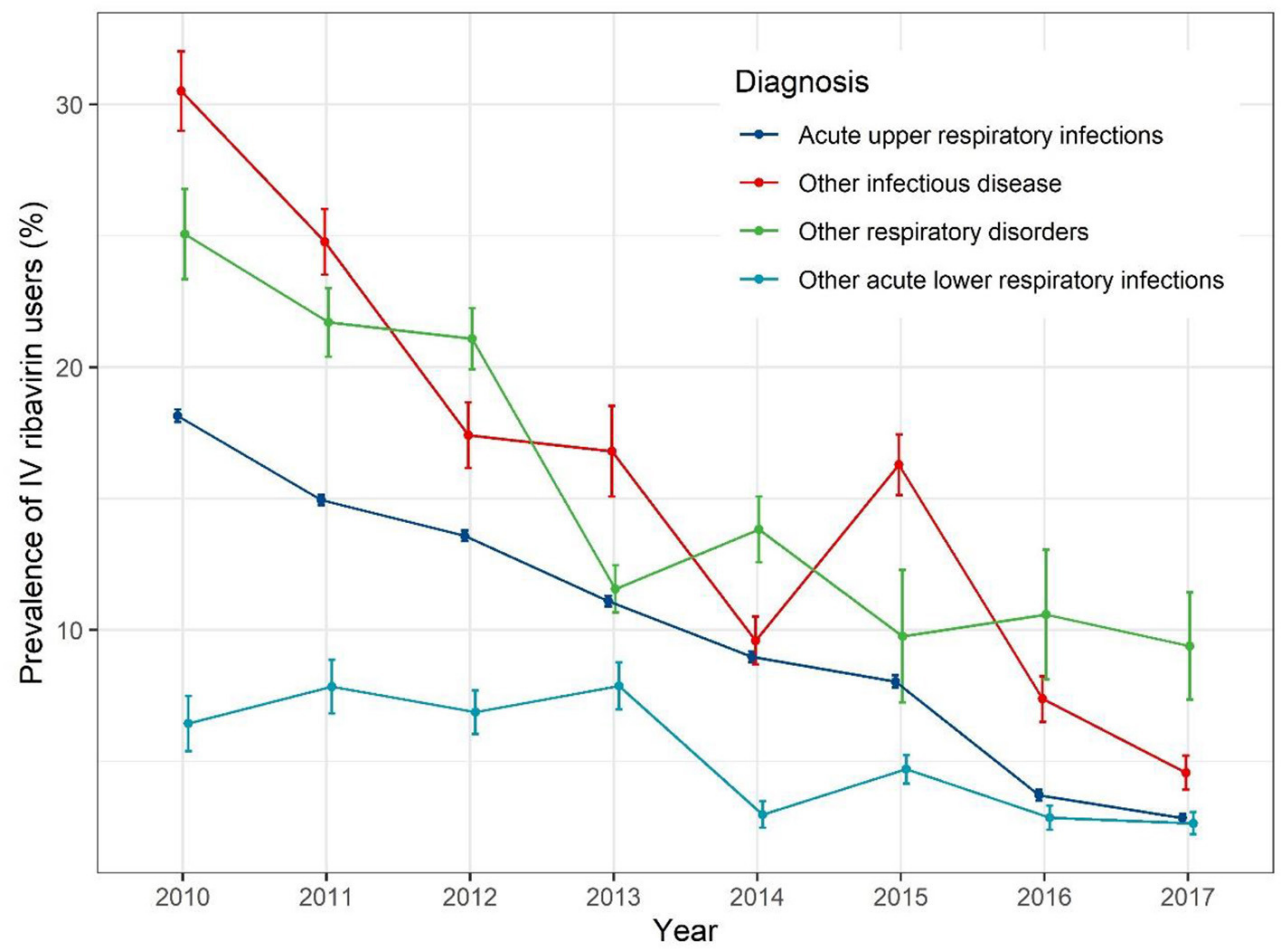
Trends of prevalence of IV ribavirin prescription for different diagnoses.

When stratified by different diagnoses (Figure 4), the IV ribavirin prescription prevalence decreased with fluctuation. For AURIs, the prevalence rate of IV ribavirin decrease gradually from 19% in 2010 to 1% in 2017.

## Discussion

To our knowledge, this is the first study to investigate the prevalence of IV ribavirin use among reproductive age adults in China. We found nearly 6% of residents had at least one prescription of IV ribavirin during the study period. IV ribavirin was commonly used among the residents aged 18–44 years in Yinzhou District during 2010–2017. And studies had raised the concerned about overuse of intravenous therapy in China (21, 22). However, IV ribavirin can only be authorized for patients with life-threatening diseases as an Emergency Investigational New Drug (EIND) applicant in USA. Andrea et al. found that 608 IV ribavirin EIND requests were made for 19 disease conditions from 1997 to 2008 *via* FDA's EIND database (2). Clinical outcome and adverse events were deficient in the report of the EIND database, so further analysis of either outcome was not feasible.

The prevalence of exposure to IV ribavirin varied by age, marital status, education level, occupation and hospital level (*P* < 0.001). Relative high prevalence were found in elder adults, unmarried residents, residents with <9 years of education, students, and farmers and unemployed residents. In addition, primary hospitals dispensed a higher proportion of IV ribavirin. A similar pattern was found in a study in the US, which is to evaluate the prevalence of exposure to antiviral medications during pregnancy in a cohort of pregnant women (23).

Our results manifest that the majority of IV ribavirin use was for AURIs. These results were different from another study, which illustrated a large proportion of IV ribavirin use EIND requests concerned adenovirus infections in immunocompromised patients. Evidence suggests that IV ribavirin may be an alternative treatment for patients with hemorrhagic fever (24, 25). In our study, there were just 4 patients with diagnosis of viral hemorrhagic fevers, whereas only one of them had a prescription of IV ribavirin. In China, treatment for pneumonia and bronchitis due to RSV is the main indication of IV ribavirin. However, a study revealed that only 1% of the patients who sought medical care for acute respiratory infections was detected positive for RSV (9). In our study, only 34 patients were tested for RSV in 2010–2017 and 6 of them were positive. Accordingly, we speculated that a large number of IV ribavirin were misused in China.

A Canadian study indicated that high-dose ribavirin is associated with a high risk of adverse events (26). According to the instruction of the intravenous ribavirin (500 mg each time, 2 times per day), we found that there were <1% of users and prescriptions of IV ribavirin were overdose for daily use. For single dose, there were about 14% of prescriptions of IV ribavirin were overdose.

The annual prevalence of IV ribavirin users decreased from 1.72% in 2010 to 0.24% in 2017 (*P* < 0.001). A general decrease of prevalence of IV ribavirin were found in different levels of hospitals. For different diagnoses, the prevalence of IV ribavirin decreased with fluctuation. Several factors may have contributed to the decrease of IV ribavirin use: First, complying with the hospital administration of prescription comment released by the National Health Commission in 2010 (27, 28), prescription evaluation and feedback for improvement by pharmacists can promote the rational use of drugs. Second, the government suggests that medical staff should pay attention to its reproductive toxicity, hemolytic anemia, and other safety issues (29).

Up to now, because of limited data on human pregnancy exposures to ribavirin, there is not enough evidence of embryotoxicity and teratogenicity of ribavirin in humans. A ribavirin pregnancy registry has been established and operated since 2003 in the United States (30). It monitors maternal-fetal outcomes of pregnant women or female partners of male patients exposed to ribavirin. The primary purpose of the registry is to evaluate the association between teratogenic risk and ribavirin exposures during pregnancy or within 6 months after treatment is stopped. Exposure may be incurred directly, when the pregnant female takes ribavirin, or indirectly, when her male sexual partner is treated with ribavirin. For registry purposes, exposure is defined as direct or indirect exposure to ribavirin during pregnancy or within the 6 months before pregnancy. Using data collected from 2003 to February 2016 in this registry study, preliminary findings do not indicate a clear signal of human teratogenicity for ribavirin (31). However, it is insufficient to draw the definitive conclusions under the current limited sample size. Despite this, patients and physicians should be aware of the potential teratogenicity of ribavirin and follow the instructions. Since there is no such pregnancy registry in China, we suggest to further investigate the risk of ribavirin among pregnant women by using linked database of birth registry and electronic medical records.

## Strengths and Limitations

Our study had several strengths. First, our study was the first to investigate the utilization of intravenous ribavirin among adults aged 18–44 in China by using a regional healthcare database in a real-world context. Second, exposure of IV ribavirin using prescription records had the advantage of avoiding recall bias. Third, the data on the demographic characteristics, hospital level, diagnosis, and prescription dosage were available, so we can delineate the prevalence across different subpopulations. Finally, these data provided a cohort which can be followed longitudinally for further investigations of safety and risk-benefit of treatment with IV ribavirin.

Limitations should be acknowledged in the interpretation of our findings. First, since our study relied on a regional healthcare database, it only covered the population of Yinzhou district of the Ningbo city which is located at eastern China, thus interpretation and extrapolation should be made cautiously. Although the cohort is geographically limited, it comprises nearly fifty thousand patients exposed to IV ribavirin in different levels of hospital which is the largest study of ribavirin use to date that we are aware of. Second, this was a descriptive study and clinical data such as laboratory results, symptoms, and prescriber qualification in the database for the drug use were not assessed.

## Conclusion

In conclusion, this study is an electronic medical records based study to investigate the utilization of intravenous ribavirin in China from 2010 to 2017. Our findings demonstrate the high prevalence of exposure to intravenous ribavirin in Chinese childbearing-aged population, compared to United States, especially among elder patients, or patients with low level of education and income, or in primary hospitals. Although the annual prevalence was decreasing over past 8 years, clinicians and pharmacists should continually avoid inappropriate use of IV ribavirin. Furthermore, we found IV ribavirin were mainly used for acute respiratory infection suggesting that large amount of IV ribavirin use were inappropriate because most of the acute respiratory infection were not caused by RSV. Given these findings, together with the limited research on the safety and effectiveness of treatment with IV ribavirin, future research is needed. This study provides a cohort with a large population and comprehensive medical records for further researches on the safety and effectiveness of IV ribavirin use.

## Data Availability Statement

The data analyzed in this study is subject to the following licenses/restrictions: Data can only be used locally. Requests to access these datasets should be directed to Hongbo Lin, lin673160@163.com.

## Ethics Statement

The studies involving human participants were reviewed and approved by Peking University Health Science Center Institutional Review Board. Written informed consent for participation was not required for this study in accordance with the national legislation and the institutional requirements.

## Author Contributions

HLi and SZ: contributed to the study conception and design. HLi and HZ: analyzed the data and drafted the manuscript. HLin, PS, and CL: critically revised the manuscript. All authors were involved in interpretation of data, read, and approved the final manuscript.

## Funding

This study was supported by the National Natural Science Foundation of China (81973146 and 72004151).

## Conflict of Interest

The authors declare that the research was conducted in the absence of any commercial or financial relationships that could be construed as a potential conflict of interest.

## Publisher's Note

All claims expressed in this article are solely those of the authors and do not necessarily represent those of their affiliated organizations, or those of the publisher, the editors and the reviewers. Any product that may be evaluated in this article, or claim that may be made by its manufacturer, is not guaranteed or endorsed by the publisher.
