# Supplementary material for: Utilization of Intravenous Ribavirin Among Reproductive Age Adults in 2010–2017: A Population-Based Study in the Yinzhou District, Ningbo City of China
Source: Front Public Health. 2021 Sep 17;9:678785. doi: 10.3389/fpubh.2021.678785 (PMC8484711; doi:10.3389/fpubh.2021.678785)
Supplement: Supplementary file 1 [file Table_1.docx]

Appendix Table 1. Prevalence of users and dispensed of IV ribavirin by Calendar Year among residents aged 18-44 years

| Year | General population | Ribavirin users | PR (%) | 95% CI |
| --- | --- | --- | --- | --- |
| 2010 | 801,667 | 13,755 | 1.72 | 1.69-1.74 |
| 2011 | 788,990 | 13,232 | 1.68 | 1.65-1.71 |
| 2012 | 775,491 | 12,384 | 1.60 | 1.57-1.62 |
| 2013 | 756,623 | 9,110 | 1.20 | 1.18-1.23 |
| 2014 | 735,697 | 6,254 | 0.85 | 0.83-0.87 |
| 2015 | 711,394 | 4,524 | 0.64 | 0.62-0.65 |
| 2016 | 687,105 | 1,764 | 0.26 | 0.24-0.27 |
| 2017 | 663,208 | 1,564 | 0.24 | 0.22-0.25 |

PR: prevalence rate, where the denominator was the total number of general population aged 18-44 years old in specific year.

Appendix Table 2. Prevalence of users and dispensed of IV ribavirin by Calendar Year among patients aged 18-44 years

| Year | Patients | | |  | Prescriptions | | |
| --- | --- | --- | --- | --- | --- | --- | --- |
|  | Ribavirin | All | PR^a^ (%) |  | Ribavirin | All | PR^b^ (%) |
| 2010 | 13,755 (28.49) | 73,229 | 18.78 |  | 24,256 (23.32) | 379,208 | 6.40 |
| 2011 | 13,232 (27.40) | 83,022 | 15.94 |  | 23,817 (22.90) | 462,822 | 5.15 |
| 2012 | 12,384 (25.65) | 86,277 | 14.35 |  | 20,786 (19.98) | 475,626 | 4.37 |
| 2013 | 9,110 (18.87) | 91,106 | 10.00 |  | 14,178 (13.63) | 429,810 | 3.30 |
| 2014 | 6,254 (12.95) | 88,458 | 7.07 |  | 9,512 (9.14) | 432,732 | 2.20 |
| 2015 | 4,524 (9.37) | 82,493 | 5.48 |  | 6,690 (6.43) | 358,135 | 1.87 |
| 2016 | 1,764 (3.65) | 79,798 | 2.21 |  | 2,494 (2.40) | 303,893 | 0.82 |
| 2017 | 1,564 (3.24) | 122,710 | 1.27 |  | 2,288 (2.20) | 412,146 | 0.56 |

^a^ PR: prevalence rate, where the denominator was the total number of patients aged 18-44 years old in specific year.

^b^ PR: prevalence rate, where the denominator was the total number of prescriptions of patients aged 18-44 years old in specific year.
